# A mushroom diet reduced the risk of pregnancy-induced hypertension and macrosomia: a randomized clinical trial

**DOI:** 10.29219/fnr.v64.4451

**Published:** 2020-06-08

**Authors:** Linlin Sun, Zhanjie Niu

**Affiliations:** Department of Obstertrics, Liaocheng People Hospital, Liaocheng, Shandong, China

**Keywords:** pregnancy-induced hypertension, non-pharmacological intervention, mushroom, preeclampsia, clinical trial

## Abstract

**Background:**

Pregnancy-induced hypertension (PIH) is a disease characterized by high blood pressure detected after 20 weeks of pregnancy, affecting approximately 10% of pregnant women worldwide. Effective strategies are imperatively needed to prevent and treat PIH.

**Methods:**

Subjects were required to consume 100 g mushroom daily from pre-pregnancy to the 20th week of gestation. The gestational hypertension and related primary and secondary outcomes of the mushroom diet (MD) group and placebo group were investigated to compare the intervention of a MD on the PIH and preeclampsia-associated maternal and child health conditions.

**Results:**

A total of 582 and 580 subjects belonging to the MD group and placebo group were included for the analysis, respectively. Compared to the placebo, the MD significantly reduced the incidence of gestational hypertension (*P* = 0.023), preeclampsia (*P* = 0.014), gestational weight gain (*P* = 0.017), excessive gestational weight gain (*P* = 0.032) and gestational diabetes (*P* = 0.047). Stratified analysis showed that the MD lowered the risk of PIH for overweighed women (*P* = 0.036), along with the percentage of macrosomia (*P* = 0.007).

**Conclusion:**

An MD could serve as a preventative strategy for lowering the risk of PIH and could control newborn birthweight while reducing comorbidities including gestational weight gain, diabetes etc.

## Popular scientific summary

- Mushroom diet could serve as a preventative strategy for lowering the risk of pregnancy-induced hypertension and may control newborn birth weight while reducing comorbidities including gestational weight gain, diabetes etc.

Pregnancy-induced hypertension (PIH) is a disease characterized by high blood pressure during pregnancy and affects about 9.4% of pregnant women in China, and 7–12% of pregnant women in other countries (1). PIH can be classified into gestational hypertension, preeclampsia, eclampsia, chronic hypertension, and eclampsia, among which gestational hypertension and pre-eclampsia are the two most common types (2). Women with PIH often face many risks, such as caesarean section, placental abruption, renal dysfunction and associated cardiovascular disease (3). PIH also affects the fetus by limited fetal growth, premature birth, abnormal fetal weight as well as health issues of the offspring, such as obesity and cardiovascular disorders, etc. (2, 4, 5). In addition, nearly 10–15% of gestational deaths are caused by preeclampsia or eclampsia in low- and middle-income countries (6). Therefore, effective strategies are imperatively needed to prevent and treat hypertensive disorders of pregnancy.

Previous studies have revealed that endothelial cell dysfunction and oxidative stress are responsible for the occurrence of preeclampsia (4, 7). Accordingly, the reduction of intracellular antioxidant levels is considered to be associated with endothelial cell dysfunction and oxidative stress. Traditional anti-hypertension drugs, such as angiotensin II receptor blockers (ARBs) or angiotensin-converting enzyme (ACE) inhibitors, are often not suitable for preconception or pregnant women. Conventional non-pharmacological treatments, such as exercise, weight loss and low-salt diets, are not recommended for pregnant women (8). For Methyldopa, although the data show that the offspring of mothers who had taken the drug have not had obvious symptoms of growth and development abnormalities; the safety concern remains for the wide usage in pregnant women (8, 9).

Mushrooms are fungi whose effectiveness in improving health has been well-documented throughout human history. China was one of the first countries to cultivate mushrooms. There are a great variety of mushrooms available in the market; these include: white button, crimini, portabella varieties, shiitake, straw, oyster, and enoki (10). Mushrooms contain various nutrients such as niacin, riboflavin, pantothenic acid, phosphorus, copper, selenium, Vitamin B12, ergosterol, and ergothioneine, some of which have been reported to be effective in the prevention and treatment of PIH (3, 6, 7, 10–12). In addition, mushrooms have many other characteristics, such as a delicious taste, relative safety, being low in calories and rich in fiber, all of which are suitable for pregnant women (11).

Here we designed and conducted a randomized clinical trial to investigate the impact of mushrooms on pregnancy-related complications. Although not well-understood, current research indicates that the pathogenesis of preeclampsia begins in early pregnancy (5), so we should intervene from an early stage of pregnancy. Therefore, in this clinical trial, subjects were required to add a certain amount of white mushroom, which is native to grasslands in North America and Europe, but now commercially and widely available in China, to the daily diet from the pre-pregnancy stage to the 20th week of gestation. Further, we tested the subjects’ gestational hypertension and related primary and secondary outcomes, aiming to investigate the effects of a mushroom diet (MD) on PIH and preeclampsia-associated maternal and child health conditions.

## Materials and methods

### Study design

This clinical trial was conducted from February 2016 to October 2018. All clinical protocols were reviewed and approved by the Institutional Review Board of Liaocheng People Hospital, and was based on the guidelines of the Declaration of Helsinki. Written informed consents were obtained from all participants prior to the commencement of the study.

### Participants and randomization

The participants were recruited mainly from the staff of the community health service center, who sought qualified female subjects via in-person interview, phone interview, as well as advertisement.

### Inclusion criteria

Given the higher risk of gestational hypertension-related diseases in primiparas, only women of childbearing age, who planned for the first pregnancy were included (6).

### Exclusion criteria

1) under 18 years old; 2) unmarried; 3) smoking; 4) already pregnant; 5) already suffering from chronic hypertension with persistent proteinuria symptoms; 6) suffering from other types of diseases, such as urolithiasis, kidney disease, thyroid-related diseases or gastrointestinal disorders; 7) HIV, Hepatitis-B, or Hepatitis -C-positive patients; 8) taking relevant contraceptive measures; 9) failure to sign a written consent, including those unwilling to deliver in a designated hospital.

After signing the informed consent, all eligible women were assigned to two groups in a 1:1 ratio randomly: the MD group and the placebo group. Participants’ randomization was achieved by an allocation concealment process through a random numbers table. The entire allocation process includes sequence generation, allocation concealment, and allocation implementation, which were completed by three different persons independently.

### Intervention

The duration of treatment is from the time of enrollment to the 20-week gestation period. For subjects in the MD group, they were required to consume at least 100 g of white button mushrooms daily, which could be cooked according to their preferences. To improve the subjects’ compliance, mushroom recipes, appropriate reimbursement and nutrition consulting service were provided to them. Healthcare specialists visited the participants on a monthly basis to gather information about the women’s mushroom-buying based on the grocery receipts. For subjects in the placebo diet group, they followed a normal diet across the study period. All subjects underwent a comprehensive examination upon enrollment and consecutive examinations at clinical trial sites every 4 weeks. Once pregnancy was confirmed, the subjects were required to be examined at least one time during the first trimester, once a month during the second trimester, once every other week from week 28 to week 36, and once a week thereafter until labor.

### Participant demographics

Demographic data and other relevant information were obtained during the first visit or retrieved from the institutional medical database. Inclusion and exclusion criteria were determined based on the discussion with obstetricians.

### Outcomes

Upon the completion of the clinical trial, the relevant information of the subjects was collected and analyzed from the hospital records. Primary outcome: gestational hypertension, the subject’s diastolic and systolic blood pressure levels were measured and recorded routinely during each hospital visit. Gestational hypertension is defined as the high blood pressure that develops after week 20 in pregnancy, when the systolic pressure ≥140 mm Hg or the diastolic pressure ≥90 mm Hg lasts for more than 4 h (4).

## Secondary outcome

1) Pre-eclampsia, defined as hypertensive disorder and proteinuria in pregnancy, which was diagnosed by professional clinical specialists; 2) gestational body weight gains, which were calculated as the body weight prior to childbirth minus the weight at the first check-up. Excessive gestational body weight gain is defined as follows: >18 kg as underweight, >16 kg as normal weight, >11.5 kg as overweight, and >9 kg as obese, categorized by the baseline body mass index (BMI) of the subjects; 3) other pregnancy complications, such as gestational diabetes mellitus (GDM), preterm delivery, and delivery type etc.; 4) birthweight: the body weights of newborns were collected from the hospital’s records.

### Statistical analysis

PIH affects about 10% women in China. We speculated that there will be a 50% reduction due to the adoption of MD. Under this assumption, a two-sample comparison (X^2^) with a 5% level of significance (α = 0.05) and a statistical power of 0.10 (β = 0.1) required a study population of 576 patients in each group. Given an approximate loss to follow-up of 8%, 622 women were initially selected in our study.

We compared the categorical variables in the baseline characteristics of the two study groups by means of a χ^2^-test. For the continuous data, a Student’s *t*-test was used for conducting statistical analysis. Further, the primary outcome and the secondary outcome in two study groups were used as clinical endpoints in a logistic regression analysis model.

## Results

### Flow of the study

**Fig. 1:**
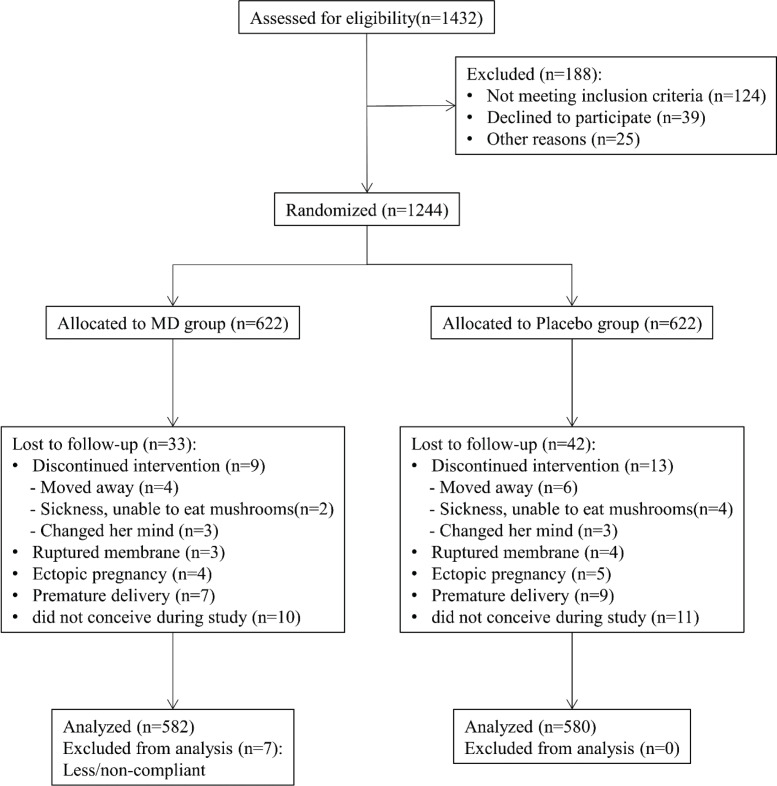
Participant flow through the study.

The schematic flow of the study is shown in Fig. 1. Initially, there were 1,432 subjects assessed for eligibility. Among them, 124 subjects were excluded according to the inclusion criteria, 39 subjects declined to participate, and 25 subjects were excluded for some other reason. The remaining 1,244 subjects were randomly allocated into the MD group and placebo group (both *n* = 622). In the MD group, nine subjects were lost to follow-up due to discontinued intervention, four subjects moved away, three subjects were unable to eat mushrooms owing to illness, and three subjects changed their minds. Further, more subjects could not participate in the follow-up due to gestational disorders including ruptured membrane (*n* = 3), ectopic pregnancy (*n* = 4), and premature delivery (*n* = 7). Moreover, there were 10 subjects who did not conceive during the study period. Preliminary analysis showed that the daily intake of mushroom was 82 ± 15 g for seven subjects, who were considered as less/non-compliant, and further excluded from the ultimate analysis. In the placebo group, there were 42 subjects lost to follow-up due to discontinued intervention (*n* = 13), moving on (*n* = 6), inability to consume mushroom (*n* = 4), change of mind (*n* = 3), as well as gestational disorders including ruptured membrane (*n* = 4), ectopic pregnancy (*n* = 5), and premature delivery (*n* = 9). Moreover, 11 subjects did not get pregnant during the study period in the placebo group. Ultimately, the characteristics of 582 and 580 subjects were analyzed for the MD group and placebo group, respectively.

### Baseline maternal characteristics of the participants during enrollment

As listed in Table 1, no significant difference was observed between the MD and placebo groups for the baseline characteristics of the participants, which include maternal age (31.2 ± 4.5 vs. 31.4 ± 4.3 years, *P* = 0.830), pregnancy BMI (22.47 ± 3.66 vs. 22.61 ± 4.01 kg/m^2^, *P* = 0.531), education years (*P* = 0.782), members of Han ethnicity (*P* = 0.684), occupational activity (*P* = 0.429), systolic blood pressure (114.3 ± 12.4 vs. 113.6 ± 13.2 mm Hg, *P* = 0.350), and diastolic blood pressure (68.5 ± 10.7 vs. 68.9 ± 9.6 mm Hg, *P* = 0.485). These data suggested that the baseline backgrounds of the participants in the two groups were very well-matched.

**Table 1:** Baseline maternal characteristics of the participants at enrollment

Maternal characteristics	Mushroom diet (MD) (*n* = 582)		Placebo (*n* = 580)		*P*-value
	Mean ± standard deviation (SD)	*n* (%)	Mean ± SD	*n* (%)	
Maternal age	31.2 ± 4.5		31.4 ± 4.3		0.830
20–24 years	22.3 ± 1.6	416 (71.5)	22.4 ± 1.5	418 (72.1)	
25–29 years	26.8 ± 1.7	141 (24.2)	26.6 ± 1.8	141 (24.3)	
≥30 years	32.6 ± 2.4	25 (4.4)	32.5 ± 2.7	21 (3.6)	
Prepregnancy body mass index (BMI), kg/m^2^	22.47 ± 3.66		22.61+ 4.01		0.531
<18.5 kg/m^2^		5 (0.9)		6 (1.1)	
18.5–24.9 kg/m^2^		487 (83.6)		480 (82.7)	
24.9–29.9 kg/m^2^		78 (13.4)		80 (13.8)	
≥30 kg/m^2^		12 (2.1)		14 (2.4)	
Education					0.782
High school or higher		178 (30.6)		175 (30.1)	
Secondary		391 (67.3)		393 (67.7)	
Primary school or lower		13 (2.1)		12 (2.2)	
Ethnicity					0.684
Han		575 (98.8)		574 (98.9)	
Other		7 (1.2)		6 (1.1)	
Occupational activity					0.429
Sedentary job		142 (24.4)		143 (24.7)	
Homemaker		112 (19.2)		110 (18.9)	
Active job		328 (56.4)		327 (56.4)	
Blood pressure, mm Hg					
Systolic	114.3 ± 12.4		113.6 ± 13.2		0.350
Diastolic	68.5 ± 10.7		68.9 ± 9.6		0.485

Data are expressed as mean ± SD, unless otherwise indicated. We analyzed continuous and nominal data by using the Student’s *t*-test for unpaired data and χ^2^-test, respectively. There are no statistical differences between groups at baseline (*P* > 0.05).

### Effects of MD on hypertension and other maternal outcomes

As illustrated in Table 2, compared to the placebo group, MD significantly decreased the incidence of gestational hypertension (48% vs. 24%, *P* = 0.023), preeclampsia (12% vs. 4%, *P* = 0.014), gestational weight gain (12.5 ± 4.5 vs. 11.8 ± 3.9, *P* = 0.017), excessive gestational weight gain (205% vs. 135%, *P* = 0.032), and gestational diabetes (32% vs. 23%, *P* = 0.047). MD in pregnancy also had a significant impact on the birthweight of newborns. Specifically, the percentage of adequate birth weight 2,500–4,000 in the MD group is 93.6%, and that of the placebo group is 87.5%, with a *P*-value of 0.013. The percentage of macrosomia (newborn birthweight larger than 4,000 g) in the MD group is 2.1%, but 7.1% in the placebo group, with a *P*-value of 0.007. No differences were seen between the groups regarding gestational age (*P* = 0.089), type of delivery including normal (*P* = 0.225), instrumental (*P* = 0.381) and cesarean types (*P* = 0.097), average birthweight of the newborn infants (*P* = 0.37), the percentage of baby with weight lower than 2,500 g (*P* = 0.468), and Apgar scores (1 and 5 min, *P* = 0.319 and 0.472, respectively).

**Table 2:** Effect of mushroom diet versus placebo on hypertension and other pregnancy outcomes

Outcomes	All (*n* = 1,062)		*p*-value
	Mushroom diet (MD) (*n* = 582)	Placebo (*n* = 580)	
Gestational hypertension, *n* (%)	24 (4.1)	48 (8.2)	**0.023**
Preeclampsia, *n* (%)	4 (0.7)	12 (2.1)	**0.014**
Gestational weight gain, kg	11.8 ± 3.9	12.5 ± 4.5	**0.017**
Excessive gestational weight gain, *n* (%)	135 (23.2)	205 (35.3)	**0.032**
Gestational diabetes, *n* (%)	23 (3.9)	32 (5.5)	**0.047**
Preterm delivery, <37 weeks, *n* (%)	44 (7.6)	47 (8.1)	0.089
Type of delivery, *n* (%)			
Normal	393 (67.6)	379 (65.4)	0.225
Instrumental	86 (14.7)	92 (15.9)	0.381
Cesarean	103 (17.7)	108 (18.7)	0.097
Newborn infants			
Birthweight, g	3,287 ± 395	3,291 ± 389	0.37
Adequate 2,500–4,000, *n* (%)	545 (93.6)	508 (87.5)	**0.013**
Low <2,500, *n* (%)	23 (4.0)	29 (5.0)	0.468
Macrosomia >4,000, *n* (%)	12 (2.1)	41 (7.1)	**0.007**
Apgar score 1 min			
≥7, *n* (%)	555 (95.3)	546 (94.2)	0.319
Apgar score 5 min			
≥7, *n* (%)	580 (99.6)	579 (99.8)	0.472

Data are expressed as *n* (%) or mean ± SD, unless otherwise indicated. We analyzed continuous and nominal data by using the Student’s *t*-test for unpaired data and χ^2^-test, respectively. Values in bold indicate *P* < 0.05 of mushroom diet group versus the placebo group.

### PIH stratified by maternal characteristics

According to previous studies, maternal age, education, and BMI have important effects on the occurrence of PIH (4, 5). In addition, exercise is also an important factor affecting the occurrence of PIH. Women with regular physical exercise have lower risk of PIH and preeclampsia. In our study, occupational activities are related to the amount of physical activity of women. Therefore, we stratified the effects of these four factors on PIH (Table 3). Briefly, participants in the age group of 20–24 years, women in the placebo group had over two times of likelihood to develop hypertension during pregnancy than the women in the MD group (odds ratio [OR] = 2.04, 95% confidence interval [CI] = 0.91–4.6, *P* = 0.023). With the endpoint as pregnancy BMI at 24.9–29.9 kg/m^2^, women in the placebo group had more than two times likelihood to develop hypertension during pregnancy than the women in the MD group (BMI 24.9–29.9 kg/m^2^, OR = 1.96, 95% CI = 0.72–3.78, *P* = 0.036). With regard to the primary school or lower education, women in the placebo group had more than two times likelihood to develop hypertension during pregnancy than the women in the MD group (OR = 1.96, 95% CI = 0.72–3.78, *P* = 0.039). With regards to sedentary job, women in the placebo group are two times more likely to develop hypertension during pregnancy than the women in the MD group (sedentary job, OR = 1.67, 95% CI = 0.90–3.16, *P* = 0.12).

**Table 3:** Pregnancy-induced hypertension stratified by maternal characteristics

Maternal characteristics	Pregnancy-induced hypertension		*P*-value
	Mushroom diet (MD) (*n* = 582)	Placebo (*n* = 580)	
Maternal age			
20–24 years, *n* (%)	17 (4.1)	32 (7.7)	**0.013**
25–29 years, *n* (%)	10 (7.1)	11 (7.8)	0.121
≥30 years, *n* (%)	2 (8.0)	2 (9.5)	0.357
Prepregnancy body mass index (BMI), kg/m^2^			
<18.5 kg/m^2^, *n* (%)	0	0	
18.5–24.9 kg/m^2^, *n* (%)	6 (1.2)	25 (5.2)	0.014
24.9–29.9 kg/m^2^, *n* (%)	3 (3.8)	6 (7.5)	**0.031**
≥30 kg/m^2^, *n* (%)	1 (8.3)	1 (7.1)	0.248
Education			
High school or higher, *n* (%)	14 (7.9)	14 (8.0)	0.462
Secondary, *n* (%)	33 (8.4)	34 (8.7)	0.520
Primary school or lower, *n* (%)	1 (7.7)	2 (16.7)	**0.023**
Occupational activity			
Sedentary job, *n* (%)	8 (5.6)	12 (8.4)	**0.025**
Homemaker, *n* (%)	8 (7.1)	8 (7.3)	0.370
Active job, *n* (%)	15 (4.6)	16 (5.0)	0.518

Data are expressed as *n* (%), unless otherwise indicated. χ^2^ analyses were used to examine statistical differences. Values in bold indicate *P* < 0.05 for mushroom diet group versus placebo group.

### Effects of MD on pregnancy outcomes based on prepregnancy BMI categories

We further analyzed the secondary outcomes related BMI in the MD and placebo groups (Table 4). Specifically, for women with normal weight, women in the placebo group have two times more likelihood to develop excessive gestational weight gain (OR = 1.93, 95% CI = 1.56–3.87, *P* = 0.043), 2.7 times more likelihood to develop gestational diabetes (OR = 2.66, 95% CI = 1.38–3.62, *P* = 0.024), and three times more likelihood to have a baby with macrosomia (OR = 2.98, 95% CI = 1.83–5.76, *P* = 0.012). For overweight women, women in the placebo group are 2.1 times more likely to develop gestational diabetes (OR = 2.11, 95% CI = 1.09–4.43, *P* = 0.082), three times more likely to have babies with weight lower than 2,500 g (OR = 2.98, 95% CI = 1.83–5.76, *P* = 0.012), and 2.5 times more likely to have babies with macrosomia (OR = 2.50, 95% CI = 1.30–4.17, *P* = 0.032).

**Table 4:** Effect of mushroom diet on pregnancy outcomes by prepregnancy body mass index categories

Outcomes	Body mass index categories							
	Underweight<18.5 kg/m^2^		Normal weight 18.5–24.9 kg/m^2^		Overweight 24.9–29.9 kg/m^2^		Obese ≥30 kg/m^2^	
	Mushroom diet (MD) (*n* = 5)	Placebo (*n* = 6)	MD (*n* = 487)	Placebo (*n* = 480)	MD (*n* = 78)	Placebo (*n* = 80)	MD (*n* = 12)	Placebo (*n* = 14)
Excessive gestational weight gain, *n* (%)	1 (20)	1 (16.7)	**88 (18.1)**	**156 (32.5)**	39 (50.0)	38 (47.5)	7 (58.3)	10 (71.4)
Gestational diabetes, *n* (%)	0	0	**8 (1.6)**	**19 (4.0)**	**4 (10.3)**	**8 (21.1)**	3 (42.9)	5 (50.0)
Preterm delivery, *n* (%)	0	1 (16.7)	37 (7.6)	36 (7.5)	6 (7.7)	8 (10.0)	1 (8.3)	2 (14.3)
Newborn infants weight, *n* (%)								
Adequate 2,500–4,000 g	5 (100)	5 (83.3)	459 (94.3)	440 (91.7)	**75 (96.2)**	**56 (70.0)**	8 (66.7)	9 (64.3)
Low <2,500 g	0	1 (16.7)	18 (3.7)	16 (3.3)	**3 (3.8)**	**10 (12.5)**	2 (16.7)	2 (14.3)
Macrosomia >4,000 g	0	0	**10 (2.1)**	**24 (5.0)**	**0**	**14 (17.5)**	2 (16.7)	3 (21.4)

Data are expressed as *n* (%), unless otherwise indicated. *P*-values were analyzed by χ^2^ analyses. Values in bold indicate *P* < 0.05 of mushroom diet group versus placebo group.

### Effect of MD on pregnancy outcomes based on maternal age

It has been widely known that age is an important factor affecting the pregnancy complications as well as newborn birthweight. Therefore, we conducted a stratified analysis of BMI for these secondary outcomes. The results were depicted in Table 5 and are as follows: For women aged 20–24 years, women in the placebo group have 1.8 times possibility of delivering babies with macrosomia than women in the MD group (OR = 3.69, 95% CI = 2.92–6.84, *P* = 0.003). For women aged 25–29 years, women in the placebo group are 1.8 times more likely to develop excessive gestational weight gain compared to women in the MD group (OR = 1.78, 95% CI = 1.04–3.22, *P* = 0.063), women in the placebo group are expected to develop gestational diabetes with 2.4 times greater chances than women in the MD group (OR = 2.35, 95% CI = 1.77–4.62, *P* = 0.018), and women in the placebo group had 1.8 times more likelihood to deliver babies with macrosomia babies than women in the MD group (OR = 3.70, 95% CI = 2.68–7.02, *P* = 0.002). For women aged >30 years, women in the placebo group had three times greater likelihood to develop gestational diabetes than women in the MD group (OR = 3.04, 95% CI = 1.98–6.26, *P* = 0.007), and women in the placebo group had more than 2.4 times of chances to deliver macrosomia babies than women in the MD group (OR = 2.44, 95% CI = 1.12–4.56, *P* = 0.015).

**Table 5:** Effect of mushroom diet on pregnancy outcomes by maternal age

Outcomes	Maternal age					
	20–24 years		25–29 years		≥30 years	
	Mushroom diet (MD) (*n* = 416)	Placebo (*n* = 418)	MD (*n* = 141)	Placebo (*n* = 141)	MD (*n* = 25)	Placebo (*n* = 21)
Excessive gestational weight gain, *n* (%)	46 (11.1)	68 (16.3)	**72 (51.1)**	**121 (85.8)**	17 (68.0)	16 (76.2)
Gestational diabetes, *n* (%)	15 (3.6)	14 (3.3)	**6 (4.3)**	**13 (9.2)**	**2 (8.0)**	**5 (23.8)**
Preterm delivery, *n* (%)	26 (6.3)	25 (6.0)	17 (12.1)	21 (14.9)	1 (4.0)	1 (4.8)
Newborn infants weight, *n* (%)						
Adequate 2,500–4,000 g	409 (98.3)	393 (94.0)	129 (91.5)	112 (79.4)	9 (36.0)	5 (23.8)
Low <2,500 g	12 (2.9)	16 (3.8)	7 (5.0)	11 (7.8)	2 (8.0)	2 (9.5)
Macrosomia >4,000 g	**5 (1.2)**	**19 (4.5)**	**5 (3.5)**	**18 (12.8)**	**2 (8.0)**	**4 (19.0)**

Data are expressed as *n* (%), unless otherwise indicated. *P*-values were analyzed by χ^2^ analyses. Values in bold indicate *P* < 0.05 of mushroom diet group versus placebo group.

## Discussion

In this study, we conducted a clinical trial spread over a period of 2 years and 8-month to explore the impact of mushrooms on pregnancy-related complications including PIH and macrosomia, a common fetal weight abnormality common in modern society. In comparison to the placebo group, our statistical results clearly showed that the MD group had a lower risk of gestational hypertension (*P* = 0.023), and preeclampsia (*P* = 0.014). In addition, mushroom ingestion also impacted the gestational weight gain (*P* = 0.017), excessive gestational weight gain (*P* = 0.032) and gestational diabetes (*P* = 0.047). Significantly, the MD in the MD group lowered the risk of macrosomia (*P* = 0.007), as well as the newborns with adequate weights (2,500–4,000 g) (*P* = 0.013) when compared to the placebo group. Given the popularity of edible mushroom in China, it is reassuring to see these results, which strongly suggested the benefits of MD on maternal complications. Moreover, pregnant women in China tend to consume food based on the traditional concept to supply better nutrition for the development of babies; our results provide valuable information for the gestational diet planning.

Medications such as ACE inhibitors and angiotensin II receptor antagonists conventionally prescribed for hypertension need to be discontinued upon pregnancy diagnosis, due to the deleterious fetal effects (13). Alternatively, there are many conventional non-pharmacological treatments including lifestyle change via weight loss, and low-salt diets, which are not recommended for pregnant women either (8). It has been suggested that maternal exercise could help prevent PIH and may control newborn birthweight while reducing the risk of comorbidities (5). However, it is not easy for pregnant women to maintain or improve their daily exercise amount. In response to the pathogenesis of hypertensive disorders such as preeclampsia in pregnancy, antioxidants such as vitamin C and E and resveratrol are provided as supplements to the diet. However, the results of clinical trials are not consistent or even on the contrary, mainly due to its poor membrane penetration (7, 9). B vitamins, such as Vitamin B12, can reduce homocysteine in pregnant women, which is thought to be related to hypertension (7). In addition, the World Health Organization recommends pregnant women to take appropriate amounts of calcium and vitamin D during the second stage of pregnancy (10). Generally, we believe that the comprehensive supplement of multiple nutrients can achieve better prevention and treatment of hypertension, with the prerequisite that these supplements are safe for pregnant women and the fetus. Mushrooms contain various nutrients, and are particularly rich in ergothioneine, a strong antioxidant that has the function of protecting endothelial cells (12). Moreover, mushrooms contain a natural antihypertensive agent, angiotensin-I converting enzyme inhibitory peptide (14). In short, the rational of the study design was in accordance with the well-documented benefits of mushrooms.

Despite the root cause of pregnancy, hypertension remains elusive; there are many studies suggested that the preeclampsia-related patho-physiological change start in early stage of gestation, whereas maternal symptoms do not manifest until mid to late pregnancy (15, 16). There are several maternal factors including maternal obesity or excessive gestational weight gain that are associated with the risk of the hypertensive disorders, in spite of the unknown causal link (17, 18). Our stratified results demonstrated that for the subjects with pregnancy BMI of 24.9–29.9 kg/m^2^, women in the placebo group had more than two times of likelihood of developing hypertension during pregnancy than the women in the MD group (*P* = 0.031). However, the effects of mushroom ingestion on the pregnant subjects with BMI < 24.9 kg/m^2^ were not significant when compared with the placebo group within the same BMI range. Clinical researchers have recommended that risk factors such as excessive gestational weight gain should be considered during prenatal care (19). This association between maternal obesity and PIH risk suggested that a healthy diet including mushroom ingestion would be a good direction to explore to reduce perinatal complications.

Since the downstream consequences of PIH have been connected to neonatal birthweight (weight >4,000 is defined as macrosomia, and weight <2,500 g is defined as low birthweight), (20) we also analyzed the effect of MD on newborn infants delivered by pregnant women with different BMIs. Particularly, for overweight women with BMI in the range 24.9–29.9 kg/m^2^, subjects in the placebo group are three times more likely to have babies with weight lower than 2,500 g (*P* = 0.012), and 2.5 times more likely to have babies with macrosomia (*P* = 0.032), compared to the percentage of subjects having babies with abnormal weight in the MD group. These results clearly supported the positive intervention of MD on the outcome of the newborn infants.

Although positive, there are some limitations to this study. First, we mainly measured the primary outcome of the subject’s diastolic and systolic blood pressure levels routinely during each hospital visit. In the early stage of pregnancy, we should have also performed routine laboratory tests, especially those associated with preeclampsia including values for platelet counts, uric acid, and creatinine, as a baseline to determine the clinical characteristics background of the participants. Secondly, different kinds of mushrooms might be taken by the subjects across the study, which have different nutrient compositions. For instance, the most commonly consumed WB mushrooms contains over 92% water, and a 100-g serving contains 3 g of protein, whereas Maitak contains 90% water, and a 100-g serving contains 1.9 g of protein (21). The variety of mushrooms could have different effects on the subjects. We could have limited the species of mushrooms while maintaining participant compliance as much as possible. Moreover, various recipes to cook mushrooms could introduce influences of the MD on the pregnancy-related complications. Due to the availabilities and personal preferences of other food ingredients, the participants may ingest more food in certain categories compared to others. Therefore, our study is also limited by using mushroom as a single variable.

To this date, a great number of preclinical and clinical studies have revealed that consumption of certain mushrooms would be beneficial in lowering the risk of cognition dysfunction (22, 23), cancer (24, 25), as well as improving weight management (11), and oral health (26). However, there is no study focusing on the potential benefits of pre-pregnancy mushroom supplementation in the prevention of PIH. While most current clinical trials and guidelines focus on the management of hypertension during pregnancy and breast-feeding, our study provided evidence to support the fact that MD could be applied to prevent or manage pregnancy-related hypertension.

## Conclusion

A MD could serve as a preventative strategy for lowering the risk of PIH and may control newborn birthweight while reducing comorbidities including gestational weight gain, diabetes etc.
